# Supplementary material for: Cyclodextrin-Mediated Cholesterol Depletion Induces Adiponectin Secretion in 3T3-L1 Adipocytes
Source: Int J Mol Sci. 2023 Sep 28;24(19):14718. doi: 10.3390/ijms241914718 (PMC10572842; doi:10.3390/ijms241914718)
Supplement: Supplementary file 1 [file ijms-24-14718-s001.zip › ijms-2560388-supplementary.pdf]

Supplementary Information

Methods

Imaging of adipocytes

3T3-L1 adipocytes were untreated (Ctrl) or treated with 4 mM MβCD for 2 h. Culture media was removed. Cells were washed once with phosphate buffer saline (PBS) and kept in PBS for imaging. A ZOE™ Fluorescent Cell Imager (Bio-Rad, Hercules, CA) was used to capture the live images.

Figure Legends

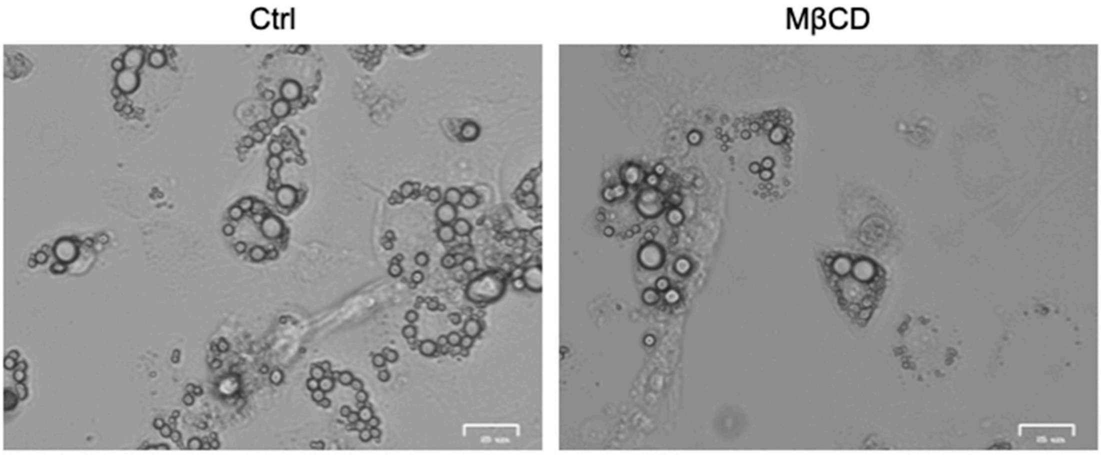

Figure S1. MβCD treatment does not affect morphology of adipocytes. Differentiated 3T3-L1 adipocytes were untreated (Ctrl) or treated with 4 mM MβCD for 2 h. Images of cells were taken. Scale bar = 25 μm.

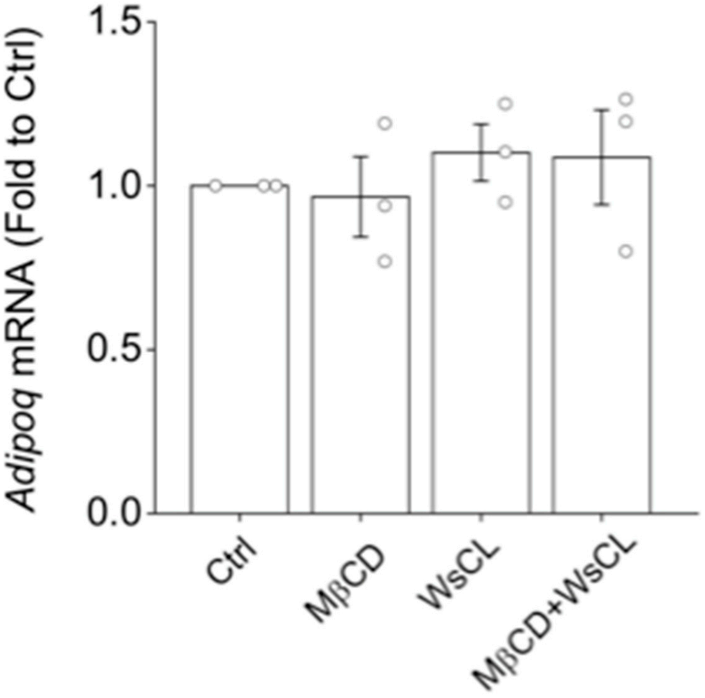

Figure S2. Adiponectin mRNA is not affected by treatment of MβCD or WsCL. Differentiated 3T3-L1 adipocytes were untreated (Ctrl), or treated with 4 mM MβCD, 250 μg/ml WsCL, or both for 2 h. Adiponectin mRNA (*Adipoq*) was measured by qPCR. Data are expressed as means ± S.E. from three independent experiments.

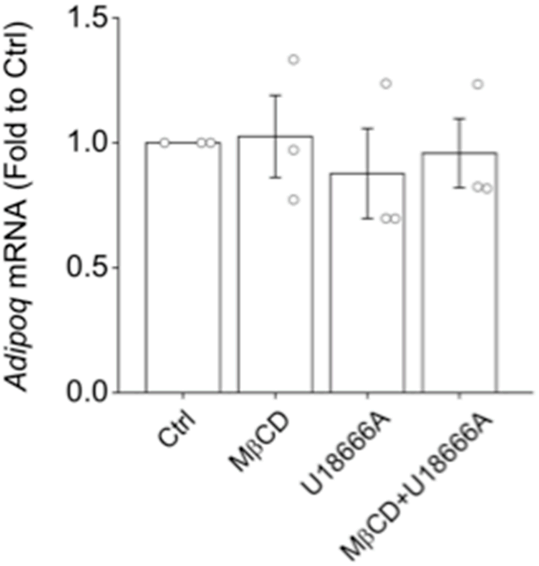

Figure S3. Adiponectin mRNA is not affected by U18666A treatment. Differentiated 3T3-L1 adipocytes were treated with vehicle (distilled water), 4 mM MβCD, 10 μg/ml U18666A, or both for 2 h. Adiponectin mRNA (*Adipoq*) was measured by qPCR. Data are expressed as means ± S.E. from three independent experiments.

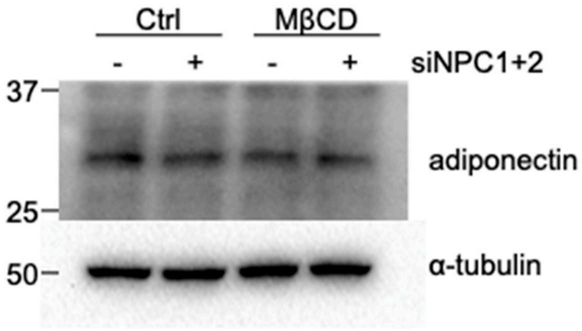

Figure S4. Depletion of NPC1 and NPC2 does not affect adiponectin protein level in adipocytes. Differentiated 3T3-L1 adipocytes were electroporated with non-targeting luciferase siRNA (siLuc) or siRNA against both NPC1 and NPC2 (siNPC1+2). Twenty-four hour post transfection, cells were untreated (Ctrl) or treated with 4 mM MβCD for 2 h. Cell lysate was subjected to Western blot analysis using adiponectin antibodies.
